# Carbohydrase Systems of *Saccharophagus degradans* Degrading Marine Complex Polysaccharides

**DOI:** 10.3390/md9040645

**Published:** 2011-04-21

**Authors:** Steven W. Hutcheson, Haitao Zhang, Maxim Suvorov

**Affiliations:** 1 Department of Cell Biology and Molecular Genetics, University of Maryland, College Park, MD 20742, USA; 2 Zymetis, Inc., 387 Technology Drive, College Park, MD 20742, USA; E-Mails: hzhang@zymetis.com (H.Z.); msuvorov@zymetis.com (M.S.)

**Keywords:** agarase, alginase, amylase, chitinase, glucanase, mannanase, pectinase, xylanase

## Abstract

*Saccharophagus degradans* 2–40 is a γ-subgroup proteobacterium capable of using many of the complex polysaccharides found in the marine environment for growth. To utilize these complex polysaccharides, this bacterium produces a plethora of carbohydrases dedicated to the processing of a carbohydrate class. Aiding in the identification of the contributing genes and enzymes is the known genome sequence for this bacterium. This review catalogs the genes and enzymes of the *S. degradans* genome that are likely to function in the systems for the utilization of agar, alginate, α- and β-glucans, chitin, mannans, pectins, and xylans and discusses the cell biology and genetics of each system as it functions to transfer carbon back to the bacterium.

## 1. Introduction

The marine environment contains a diverse collection of complex polysaccharides (CPs) as all organisms in this environment produce them. For autotrophs and heterotrophs, whether prokaryotic or eukaryotic, these polymers are associated with proteins and membranes, extracellular polysaccharides, structural elements of the cell wall, and/or storage forms of carbon [1]. They can be as simple as starches formed of α-1,4-linked glucose or as complex as the mixed polymers present in what are traditionally known as hemicellulose (e.g., xylans) or pectin. Many of these polymers have industrial applications. As few of these polymers accumulate in the marine environment, mechanisms must exist in each marine habitat to mineralize them through enzymatic degradation and metabolism.

*Saccharophagus degradans* 2–40 (*Sde*2–40; formerly *Microbulbifer degradans* 2–40) is a rod-shaped bacterium with a salt requirement typical of marine bacteria and capable of processing many CPs to their elemental sugars or sugar derivatives [2]. This aerobic, γ-subgroup proteobacterium of the *Alteromonadales* group was isolated from decaying saltwater marsh grass, *Spartina alternaflora*, in a marine estuary [3–5]. It is a versatile saprophyte that can decompose whole plant material in monoculture and expresses multi-component enzyme systems to degrade at least 10 different CPs [2–7]. *Sde*2–40 is unusual in its ability to utilize CPs of algal, higher plant, fungal and animal origin as sole carbon and energy sources. *Sde*2–40 is able to grow using agar, alginate, cellulose, chitin, α- and β-glucans, galacto-/gluco-mannans, various xylans, citrus pectin or laminarin as the primary carbon and energy source. This bacterium is also able to produce polyalkanoates from these polysaccharides [8,9].

The unusual character of this bacterium was further revealed by the genome sequence [10]. Based upon the genome annotation, this bacterium was predicted to devote a large portion of its genome to the processing of complex polysaccharides. Gene models were identified to produce enzymes containing at least 132 glycoside hydrolase domains spread among 42 families, 37 glycoside transferases, 33 polysaccharide lyases and 13 carbohydrate esterases [11]. In addition, many of the above deduced enzymes carry homologs to carbohydrate binding domains (CBMs) that function in the reversible adsorption of the host enzyme to their substrate or associated carbohydrate polymer [12]. This bacterium is annotated to express 143 homologs of CBMs distributed among 20 families [10]. These catalytic and binding domains are linked in unusual combinations to form the modular carbohydrases expressed by this bacterium. Multiple carbohydrase systems, in turn, were revealed by the genome sequence to match the observed degradative abilities of this bacterium. This review summarizes the properties of many of this bacterium’s annotated or verified carbohydrases and extends the observations of Weiner *et al.* [10]. Each system is described from a cell biology and genetic view with new genes and likely or known cellular location of each component described.

## 2. The Agarolytic System

Agar is an agarocolloid gel formed of unsubstituted and substituted agarose polymers [1]. It is a common cell wall constituent of many red algae (Rhodophyta) [13]. Up to 70% of the algal cell wall can be agar polymers. The remaining material consists of other galactans and embedded xylan and cellulose microfibrils. The base polymer of agar is agarose that is composed of repeating neoagarobiose units (3,6-anhydro-l-galactose-α-1,3-d-galactose) joined by β-1,4 bonds that forms a helix in aqueous environments. The galactose moieties of the repeating neoagarobiose units can be methylated, pyruvated, sulfonated or glycosylated to form various substituted derivatives with different gelling and solubility characteristics.

*Sde*2–40 is capable of rapid growth on agars and agarose as the dominant carbon source and produces multiple agarases [7,14]. The mechanism by which *Sde*2–40 degrades agar employs five β-agarases, designated Aga50A, Aga16B, Aga86C, Aga50D and Aga86E [15,16] and a neoagarobiose hydrolase Aga117F [17]. These agarases are modular and retain homologs of glycoside hydrolase families associated with agarase activity, such as GH16, GH50, GH86 and GH117 (Table 1). Aga16B was unequivocally demonstrated to be a freely secreted *endo-*β-agarase with a GH16 domain that rapidly degraded agar and agarose to neoagarotetraose [15]. The identification of Aga50D as a freely secreted agarase was originally based upon conserved sequence features but directed expression of the cloned gene showed it to be an *exo-*lytic agarase releasing neoagarobiose directly from agar [16]. Aga86E shares sequence similarity to several GH86 agarases and the purified enzyme almost specifically releases neoagarobiose from agarose consistent with exolytic degradation of agarose polymers [15].

A feature of Aga16B and Aga86E is the inclusion of multiple CBM6 domains [15]. CBM6 is involved in the binding of the host enzyme to its substrate [20]. The CBM6 modules found in Aga86E and Aga16B form a distinct subclass within the large CBM6 family [15,18]. Five amino acid residues are strictly conserved among CBM6s associated with agarases [20]. As expected from the modularity of these agarases, deletion of the CBM6 domains did not obviously affect the catalytic activity of either enzyme as the catalytic GH16 and GH86 domains are functional independently of other domains. These CBM6 domains did increase the affinity of these enzymes for their substrate and one of the CBM6 modules (Aga16B-CBM6-2) binds to the nonreducing end of agarose polymers [18].

Two cell-associated agarases, Aga86C and Aga50A, are also produced by this bacterium [15,21]. Aga86C is present in an 85 kDa agarolytic fraction of the bacterium. Aga50A, when expressed individually in *E. coli*, enabled the slow pitting of agar [19]. One explanation for the low apparent agarase activity of Aga50A is that this enzyme is also an *exo-*enzyme producing neoagarobiose like Aga50D. Both contain an amino acid sequence at their *N*-termini known as a lipobox [15]. This is significant because lipoboxes are associated with acylation after secretion and subsequent attachment of the host protein in the outer face of the outer membrane [22–25].

The remaining component of the *Sde*2–40 agarolytic system is a neoagarobiose hydrolase that converts the neoagarobiose released by the activity of the β-agarases to galactose and 3,6-anhydro-α-galactose. This activity was predicted to be produced by *Sde*2–40 [15] and shown to be present by metabolic profiling [21,26]. The enzyme was identified as a GH117 enzyme [11,17]. Interestingly this enzyme lacks an obvious secretion signal, suggesting it is cytoplasmic. The source gene for this enzyme is part of an apparent operon that is divergently expressed from Aga86E, suggesting they may be under common regulation.

Using these data, a model for agarose degradation by *Sde*2–40 can be assembled. Irrespective of the activity, these agarases appear to be coordinately expressed as the activities are only observed during growth on agar or agarose [7,14,21,26]. The major secreted endoagarase of *Sde*2–40 appears to Aga16B. The resident CBM6 domains may play a role in attachment of this enzyme to algal cell walls to minimize diffusion of the enzyme and could also function to destabilize the cell wall polymers. The surface-associated Aga86C may function in a similar capacity to produce neoagarooligosaccharides. Both enzymes would increase accessibility of the *exo-*acting enzymes to their substrate. Neoagarobiose would be produced by the activity of secreted Aga86E, Aga50D, and possibly the cell-associated Aga50A. The observation that neoagarobiose hydrolase is cytoplasmic indicates that this bacterium imports the released neoagarobiose. A candidate sugar transporter is divergently expressed from *aga86C*, again suggesting common regulation, and like other co-localized agarase genes, has its strongest homolog in *Pseudoalteromonas atlantica*. This transporter is designated here as AgaT as a candidate neoagarobiose transporter. Once in the cytoplasm, neoagarobiose would be converted to galactose and 3,6-anhydro-α-galactose by the activity of neoagarobiose hydrolase. The released galactose is most likely metabolized by the Leloir pathway as the enzymes for the other metabolic pathways of galactose are missing in the genome annotation [26] (although there are two candidate tagatose 1,6-P aldolases in the genome annotation). The 3,6-anhydro-α-galactose appears to be reduced to fucose and then metabolized to triose phosphate [26].

## 3. The Alginolytic System

Alginic acid is a viscous, high molecular weight polymer composed of β-1,4-linked stretches of β-d-mannuronic acid (M) and α-l-guluronic acid (G) [27]. These sugar derivatives are C5 epimers of each other. Alginic acid is found in the cell wall of the brown seaweeds (Phaeophyceae) [1] where it is believed to function as an intercellular skeletal matrix [28]. Alginate, the salt of alginic acid, comprises about 60% of the cell wall mass of *Fucus distichus* [29]. Alginate is also produced by two bacterial families, *Azotobacteriaceae* and *Pseudomonadacease* [30] as an extracellular polysaccharide and is a major component of many biofilms.

Alginate is degraded by a group of enzymes known as alginases [30–33]. Alginases are usually polysaccharide lyases (EC 4.2.2.-) acting on a wide range of naturally acidic polysaccharides and catalyze the β-elimination of the 4-*O*-linked glycosidic bond forming unsaturated uronic acid-containing oligosaccharides [30,33,34]. This depolymerization of alginate causes the formation of a double bond between the C4 and C5 of the six-carbon ring. Both *endo*- and *exo*-acting alginate lyases have been identified, ultimately releasing 4-deoxy-l-*threo*-5-hexosulose uronate from the non-reducing terminus [30,33].

*Sde*2–40 is able to grow on sodium alginate as a sole carbon source [7,35,36]. Thus, a pathway for degradation, transport and metabolism of alginate must exist. Consistent with this prediction, *Sde*2–40 appears to produce an array of alginate lyases with Alg6F as the key example (Table 2). These enzymes include polysaccharide lyase domains PL6, PL7, PL14, PL18. With the exception of Alg14M, most are annotated as poly (β-1,4-d-mannuronate) lyases. Many of these enzymes also include CBM16 and CBM32 domains as well. Some of these enzymes carry a FA58C domain that is a less defined CBM. Like other carbohydrase systems, five enzymes also include a lipobox suggestive of a surface localization. The apparent redundancy in the system could explained by: (1) substrate specificities of the enzymes; (2) *endo vs. exo* activity of the enzymes; and (3) possible differential regulation of the source genes by different substrates.

Degradation of polymeric alginate obviously occurs outside of the bacterium because all alginate lyases thought to be produced by this bacterium have secretion signals and the bacterium lacks a mechanism to import alginate. Thus, the bacterium must have mechanisms to import the released 4-deoxy-l-*threo*-5-hexosulose uronate. After import, the 4-deoxy-l-*threo*-5-hexosulose uronate could be converted to 2-dehydro-3-deoxygluconate by an isomerase, phosphorylated by a kinase and then cleaved to produce pyruvate and triose phosphate [30,33]. Putative enzymes to carry out these activities have been identified (Table 2). Interestingly, the candidate kinase is in an apparent operon with homologs to dicarboxylate transporters (DctM, DctQ and DctP) that might function in the importation of 4-deoxy-l-*threo*-5-hexosulose uronate. In addition, there is a divergently expressed GntR homolog that could function in the regulation.

## 4. The α-Glucanases

α-Linked glucans are ubiquitous polymers that include starches, glycogens, and pullulans. Starch is formed of amylose which is essentially unbranched α-1,4 glucan, and amylopectin is based upon amylose with an α-1,6 linkage approximately every 30 glycosyl units to initiate a new stretch of amylose. Glycogen is like amylopectin but the frequency of branching via α-1,6 linkages is higher. Pullulan is formed of maltotriose (3 α-1,4-linked glycosyl units) joined by an α-1,6 linkage. α-Glucans are easily utilized for metabolism due to its easily digestible nonplanar structure with α-1,4-linked glycosyl units forming a helix in solution. Branching further disrupts this structure. Starches are commonly found in algae and plants [1]. In addition to animal sources, some bacteria can also produce and accumulate glycogen as a storage product.

Degradation of amylose by bacteria usually involves α-amylases that predominantly release maltotriose, maltose (glucose-α-1,4-glucose) and some glucose. The better-known β-amylases produced by many other organisms specifically release maltose. α-Glucosidases cleave the remaining glycosyl bonds to release glucose. A third family of enzymes that include pullalanases hydrolyzes the α-1,6 bond.

Mixed starches support the growth of *S. degradans* [7]. Thus, like for the other growth-supporting carbohydrates, this bacterium can be predicted to express the enzymes to break down this material. A review of the genome annotation of *Sde*2–40 indicates the presence of a complete system to hydrolyze α-linkages between glucan units (Table 3). Three freely secreted α-amylases were identified in the genome annotation that contained GH13 domains and exhibit end-to-end similarity to known α-amylases. In contrast to many other secreted carbohydrases in this bacterium [10], these enzymes lacked any obvious CBMs. In addition, there is a starch binding protein with a CBM20 domain but no obvious catalytic domain. It is unclear whether this protein forms an association with the amylases or functions independently to destabilize starch. Three secreted α-glucosidases are produced that contain GH97 domains. The role of these enzymes in starch degradation by this bacterium has not been established.

Debranching of amylopectins/glycogen and the release of maltotriose from pullulan appear to involve a surface-associated pullulanase and its helper as homologs are present in the genome. Both proteins include a lipobox consistent with acylation and surface association. In addition, two amylases (Amy13D and GlyNAX) also carry lipoboxes. Only a GH13 α-glucosidase (Gly13E) and a sucrose phosphorylase (Suc13F) appear to be cytoplasmic. The presence of a strong candidate sucrose phosphorylase in the cytoplasm argues that this bacterium must have a mechanism to import dimeric sugars.

Overall, it would appear that since most enzymes to convert starch to glucose are secreted or surface associated, the activities of these enzymes combine to form glucose outside of the cell. Assuming they are expressed and functional, these enzymes would release glucose for importation. Import of external glucose most likely involves a sugar transporter and glucokinase. Genes 904 and 1018 are annotated to encode homologs of a glucokinase. In addition gene 1017 is divergent expressed from 1018 and encodes a glucose/galactose transporter. Metabolism of the imported glucose is predicted to occur by the Entner-Douderoff pathway as genes for the diagnostic enzymes of this pathway are present in the genome and the physiology of this bacterium is similar to pseudomonads that typically use this pathway.

## 5. The Cellulolytic System

Globally abundant cellulose is formed of linear β-1,4-glucan that assembles into paracrystalline structures in water [1,37]. It is formed by autotrophs of many taxonomic classifications as a component of their cell wall. Oomycetes can also form cellulose as well as several groups of bacteria. Degradation of cellulose involves *endo-* and *exo-*glucanases that hydrolytically form cellobiose and cellodextrins that are converted to glucose by the activity of β-glucosidases.

*S. degradans* is well established as a cellulolytic bacterium and the enzymes produced by this bacterium are described in Table 4 [2,6,7,10,38–41]. Analysis of the genome led to the demonstration that *Sde*2–40 secretes at least 15 β-1,4-endoglucanases, three of which have been reported to be processive endoglucanases (Cel5G, Cel5H and Cel5J) that appear to substitute for the cellobiohydrolases apparently absent in this system [39]. This, however, has not been independently verified and an alternative activity for Cel5H, and presumably Cel5G and Cel5J, has been proposed [42]. Three other enzymes of the system are likely processive enzymes [2,40]. These include Cel5A in which one of the GH5 domains is in the same phylogenetic clade as those of the processive GH5 enzymes and the GH9 enzymes that are processive in some other bacteria [41]. Additional enzymes to what was originally proposed [10,38], such as Gly5L and Gly5M, also appear to be part of the cellulolytic system of this bacterium [41]. These enzymes are glucanases and their parent genes are induced by cellulose. Thus, it would appear that the 12 GH5 enzymes, the GH6 enzyme and two GH9 enzymes are endoglucanases. As reported previously, most of the secreted enzymes include one or more CBM6 or CBM2/10 [38]. Some of the endoglucanases carry lipoboxes indicative of cell surface association. All of these secreted glucanases have pH optima near neutrality and are salt tolerant [39,40].

Cellobiose produced by the activity of the classical and/or processive endoglucanases appears to be metabolized by two pathways [40]. Cytoplasmic Cep94A catalyzes the phosphorolytic cleavage of cellobiose to form glucose 1-phosphate and glucose. The resulting glucose 1-phosphate would be converted to glucose 6-phosphate by the activity of a phosphoglucomutase. This is likely an energy conservation step during periods of nutrient limitation as an ATP is not consumed by phosphorolysis or in the subsequent isomerization of the glucose 1-phosphate to glucose 6-phosphate. The rate of cellobiose phosphorolysis appears to be 25% the rate of hydrolysis during rapid growth on cellulose. Hydrolysis involves five β-glucosidases. All enzymes functioning in the conversion of cellobiose to glucose or glucose phosphate were cell-associated with Cep94A, Bgl1A and Bgl1B cytoplasmic and Ced3A, Ced3B and Bgl3C exported and attached to the outer membrane, presumably by acylation. Since the cell contains a cytoplasmic cellobiose phosphorolase that is predicted to account for a large fraction of the cellobiase activity in *Sde*2–40 [40], presence of a cellobiose transporter in the system seems likely. Cep94A is produced from the *cep94A* gene in an apparent operon with a putative sugar transporter, but there is insufficient information at the present time to predict function of this apparent transporter.

Genes of the cellulolytic system are regulated by their substrate. For selected genes of the cellulolytic system, qRT-PCR has been used to identify gene sets with similar patterns of expression. As predicted from previous biochemical studies on this bacterium [7], there was a high degree of specificity to the gene induction observed. Presence of microcrystalline cellulose in glucose-deficient growth media induced expression of all of the annotated cellulase genes. Three distinct expression patterns were detected [41]. The expression of the genes for some cellulases, such as Cel5A, was induced 2–10 fold within 2 h and then expression remained relatively constant thereafter. A larger subset with Cel5H as the example was induced (>500-fold) 4–10 h after the nutritional shift but then expression was reduced by an order of magnitude at 24 h. A third group that includes Cel5I exhibited the highest average induction but only after 24 h. These distinct patterns of expression indicate that at least part of the apparent redundancy in enzymes (e.g., the 15 endoglucanases) may be due to their independent regulation by distinct transcriptional factors [2]. Each pattern of expression would represent a set of co-regulated genes responsive to a specific cellulose-linked regulatory system. If verified, it will be interesting to see what other activities are part of each regulon.

## 6. The Chitinolytic System

The second most abundant polysaccharide in the environment is chitin formed of poly β-1,4-*N*-acetylglucosamine [43]. It is found in the cell walls of fungi, the exoskeletons of arthropods and diatoms, and the feeding structures of some mollusks and cephalopods. Metabolism of chitin can be similar to that of cellulose with external degradation of the polymer to soluble chitooligosaccharides and subsequent processing to *N*-acetyl-glucosamine [44]. A chitin binding protein is essential for the process [45]. *N*-acetyl-β-glucosamine is then imported, deacetylated and deaminated to form fructose 6-phosphate. Alternatively the polymer can be deacetylated externally to form chitosan and then cleaved by chitosanases.

*S. degradans* produces a chitinolytic system that has been partially characterized by genome annotation, molecular cloning, and biochemical characterization of purified products [44,46,47]. This bacterium secretes the endochitinases Chi18A and Chi18C, the chitodextrinase Cdx18A as well as a chitin binding protein (Table 5). The released chitodextrins can be converted to chitobiose by the secreted Cdx18A and the surface-associated Chi18B. Chi18B is an interesting enzyme in that it has two GH18 domains that are separated from a lipobox and each other by polyserine domains [47,48]. With the apparent cell surface attachment as a reference point, the distal GH18 domain is an *endo-*acting domain whereas the proximal GH18 is an *exo-*acting enzyme. The juxtaposition of these domains would place production of chitobiose by this enzyme directly at the surface of the cell where it could enter the periplasm via by outer membrane porins. There is also one surface-associated *N*-acetyl-glucosaminidase (Hex20A) that could convert the externally produced chitobiose and chitodextrins to *N*-acetyl-β-glucosamine as well. In addition, there is an apparent periplasmic form of this enzyme that could convert periplasmic chitobiose and chitodextrins to *N*-acetyl-β-glucosamine. The *N*-acetyl-β-glucosamine produced by the activity of either enzyme would be imported into the cytoplasm by a NagE homolog, an inner membrane transporter, and converted to fructose 6-phosphate by the remaining Nag system (Table 5).

## 7. The Laminarinase System

Laminarin is a storage polysaccharide found in brown algae [1]. It is primarily composed of β-1,3-linked glucosyl units with occasional β-1,6 linkages. Overall, laminarin is considered to be similar in structure to amylopectin. Mannitol has also been reported in this polymer.

*Sde*2–40 grows on laminarin and both laminarinase and amylase activity can be detected under these conditions [7]. The bacterium is annotated to produce 8 candidate laminarinases [10]. With the exception of Lam81A, all carry GH16 domains (Table 6). Six of the laminarinases appear to be freely secreted. Three of these carry CBM6 domains. Gly16H, a likely laminarinase, has a CBM32 domain. Lam16B, Lam16D and Gly16H all carry at least one CBM-like FA58C domain. The laminarinodextrins produced by the activity of these enzymes are likely to be converted to glucose by the activity of one or more β-glucosidases described as part of the cellulolytic system. Thus further metabolism of laminarin is would be similar to that of cellulose. Like each of the previous systems, three enzymes were found to carry lipoboxes at their amino termini, suggesting they are surface-associated through acylation.

Degradation of the β-1,6 branches in laminarin and the laminarin-associated mannitol by this bacterium has not been established. There are genes for two apparently acylated β-1,6-glucanases in the genome of this bacterium (Table 6). Two cytoplasmic mannitol dehydrogenases are also annotated in the 2–40 genome (941 and 1241). Genes for both dehydrogenases are located within apparent operons with genes predicted to encode glucuronate isomerases. There is also a mannitol/fructose type PTS system produced by this bacterium as well (genes 3180–3182). Thus, this bacterium is predicted to have a mechanism to debranch laminarin and to metabolize whatever mannitol might be associated with laminarin.

## 8. The Mannanase System

Glucomannan is β-1,4-mannose mixed with β-1,4-glucose with slightly more mannose content than glucose. Galactomannan is β-1,(3)4-mannose with at least 5% α-1,6-galactose. Mannans are primarily found as glucomannans that can be a constituent of red algal cell walls and as galactomannans found in some leguminous seeds and fungi [1].

*Sde*2–40 is able to utilize both glucomannan and galactomannan as the primary carbon source for growth [49], thus indicating that this bacterium produces the enzymes to metabolize this polymer. The bacterium is annotated to secrete an *endo-*acting β-1,4-mannanase (Man5O) and an *exo-*acting β-1,4-mannosidase (Man5N) ([10]; Table 7). The endomannanases Man5P and ManR and the exomannosidases Man5Q and Man26A are predicted to be on the cell surface due to the presence of a lipobox. Both a mannanase and a mannosidase appear to be cytoplasmic. The reason for this is not clear. The glucan component of mannan is likely degraded by the cellulolytic system.

## 9. The Pectinolytic System

Pectic components are primarily composed of α-1,4-galacturonan with dispersed α-1,2-rhamnose residues [50]. Side chain polymers composed arabinan or arabinogalactan can be present. Three pectic polysaccharides, homogalacturonan (HG), rhamnogalacturonan-I (RG-I) and substituted galacturonans (rhamnogalacturonan-II), have been identified. HG is methylated α-1,4-d-galacturonate. RG-I has α-1,2-rhamnose alternating with α-1,4-d-galacturonate in the backbone polymer. Some of the rhamnose residues can be substituted at C-4 with linear and branched α-l-arabinofuranosyl and/or β-d-galactopyranosyl residues. RG-II is composed of 1,4-linked α-d-galacturonate substituted at C2 with non-saccharide or octasaccharide side chains and different disaccharides can be attached at C-3. These pectic compounds can comprise a major fraction of algal cell walls [1].

Degradation of pectic polymers requires pectin esterases to remove methyl groups creating methanol in the process. Pectate/pectin lyases catalyze elimination reactions at the non-reducing end of the polymer to release 4-deoxy-α-d-mann-4-enuronosyl residues. Polygalacturonase and rhamnogalacturonanase cleave the polymer hydrolytically producing galacturonic acid and rhamnose. Both lyase and hydrolytic mechanisms can be employed by the same strain for degrading pectins [51].

*Sde*2–40 is highly pectinolytic and is able to utilize neutralized citrus pectin as the primary carbon source during growth [7]. Pitting is observed on pectin-based gels consistent with the secretion of enzymes to degrade pectins and the utilization of released sugar derivatives. As with all other CPs, processing of pectin occurs external to the cell. This bacterium secretes a number of PL1, PL3, PL10 and PL11 lyases to degrade pectin polymers (Table 8). Many of these probable lyases carry CBM6 and CBM35 domains and some carry CBM2 domains. Complementing the lyases are GH105 hydrolases. In addition, there are a number of arabinofuranosidases and galactosidases that could function in the degradation of pectin as well (Table 9). All annotated pectin esterases (pectin methyl esterases) appear to be surface-associated as they carry lipoboxes at their amino termini. This suggests that the secreted lyases act on methylated pectin to release pectin fragments. There are additional lyases and hydrolases with lipoboxes as well, indicating that conversion of released pectin fragments to their constituent sugars and sugar derivatives likely occurs at the cell surface. Clearly this bacterium produces many pectinolytic enzymes. Differences in substrate specificity (HG/RG-I/RG-II)) and regulation as well as localization may account for the deduced redundancies in enzyme activities.

## 10. The Xylanolytic System

Xylans are β-1,3- or β-1,4-linked xylose that can be modified to include acetyl groups, arabinose and methylated glucuronate. Included with this polysaccharide can be arabitans composed arabinose with various linkages and arabinogalactans with α- and β-linkages [1]. In addition to their structural roles in the hemicellulose component of higher plant cell walls as would be found in marine and estuarine grasses [52], xylans can substitute for cellulose in the cell walls of some siphonous green algae [53,54] and red algae [55].

The variation among the constituent linkages in the polymers of the xylans, arabitans and galactoarabitans requires a diverse collection of enzymes to release the constituent sugars and sugar derivatives, such endoxylanases and xylosidases to produce xylose. Galactosidases, glucosidases, glucuronidases and arabinofuranosides would release galactose, glucose, glucuronate and arabinose, respectively. Acetoxylan esterase would deacetylate xylan backbones.

*Sde*2–40 can utilize xylans from terrestrial sources as the primary carbon source for energy and growth [7]. This bacterium, however, does not seem to be able to utilize arabinogalactans well [49], but it can grow on the constituent sugars [2]. A review of the genome annotation, metabolic profiling and the biochemical activities of selected genes indicates that this bacterium produces all of the enzymes to degrade and utilize xylan and arabinogalactan of marine and terrestrial origin [6,10,49,56]. This bacterium produces GH10 and GH11 xylanases [19,56] as well as the enzymes to remove backbone modifications (Table 9). In these cases, the xylanases appear to be freely secreted as >90% of the activity produced by this bacterium is present in culture filtrates [49]. Most of the enzymes to remove backbone modifications have lipoboxes suggestive of surface attachment. Only a few hypothetic esterases together with a candidate arabinofuranoside and glucuronidase have the properties of cytoplasmic enzymes.

Thus it can be predicted that depolymerization of xylans occurs external to the cell, mostly through the activity of freely secreted enzymes. Removal of modifications can occur at the source or on the cell surface. Ultimately, the released xylose, glucose, glucuronate, galactose, and arabinose are imported into the cell. This presumably involves outer membrane porins and cell membrane transporters for these sugars that have yet to be identified.

## 11. Concluding Remarks

Although the native habitat for *S. degradans* has not been established, its physiology is consistent with this bacterium being of marine origin. As such, this bacterium faces the issue of how to obtain subsistence in light of two problems. The bacterium does not import any of these minimally soluble CPs. Instead it secretes enzymes to solubilize the CP first. Any enzyme that it secretes, however, has the potential to diffuse away in the marine environment, and therefore, be lost. Similarly, recovery of any solubilized CPs faces the same difficulty. Solubilized CPs have the potential to diffuse away as well. Thus, the bacterium would needs to minimize the loss of the carbohydrases it secretes and to maximize the recovery of sugars and sugar derivatives to use as carbon and energy sources. Three features of the carbohydrase systems of *S. degradans* appear to address these problems: (1) Expression of the genes for a carbohydrase system are specifically induced by contact with their substrate; (2) the structural properties of the secreted carbohydrases favor adsorption to their substrate; and (3) the sugars or sugar derivatives derived from CPs are generated at the cell surface to maximize their uptake. This insures the maximum economy of use for each substrate CP.

In this bacterium, carbohydrases are produced on a “per need” basis as the carbohydrase systems are subject to tight genetic regulation. The degradative system for a specific polymer is only expressed in the presence of that polymer [7]. This regulation occurs at the transcriptional level [41]. As the CPs are insoluble and generally found in cell walls or as carbon storage granules, it seems reasonable to predict that the bacterium first adsorbs to the surface of these materials. Surface-associated cadherin domains might be an example of this capacity [57,58], but colloidal activity could function this way as well. To sense adsorption to a suitable substrate, a mechanism must exist to produce a signal molecule specific to that polymer. The simplest way to do this is through the activity of basally expressed sentinel enzymes. The cellodextrins produced by the activity of basally expressed glucanases and activate production of cellulases are one example of this phenomenon [41].

The bacterium must also have at least one mechanism to perceive the signal molecule. This argues for the involvement of a regulatory system to activate expression of contributing genes. For example, the cellodextrins produced by the basally expressed glucanases induce transcription of the genes in the cellulolytic system of this bacterium. Interestingly, as many as three regulatory systems could function in this process. Each regulatory system would have its own transcriptional factor. Understanding what genes are co-expressed in this bacterium as part of the regulon for each cognate transcriptional factor could help understand how to digest specific carbohydrates and raw biomass.

The localization of the carbohydrases of each system provides a plausible explanation for how CPs are used as food. Each system involves secreted enzymes to depolymerize their target CPs as the enzymes have easily recognized type II secretion signal sequences. These secreted enzymes appear to be adapted to the marine environment as they do not function well at the acidic pH’s typical of many terrestrial systems, but instead, require the more neutral environment of seawater. In addition, the activities of these secreted enzymes seem to be tolerant to salt concentrations as high as 5%.

Another unifying feature of these secreted carbohydrases is the inclusion of CBMs. The secreted enzymes many times carry one or more CBMs joined to the catalytic domains by flexible linkers. These CBMs likely assist in the adsorption of secreted enzymes to their substrate. Thus, the adsorption modules function to minimize the loss of the host enzyme through diffusion. Since adsorption occurs independently of catalytic activity, these enzymes would still be able to solubilize their substrate polymer. Loss of secreted carbohydrases would be minimal as they would only be produced when the bacterium is adsorbed to that substrate and the secreted carbohydrases would be bound to their substrate through their CBMs to limit diffusion. For those enzymes that lack obvious CBMs, it will be interesting to see if they interact with proteins that do carry CBMs as part of multimeric complexes.

Interestingly, the secreted carbohydrases are likely to only partially depolymerize their substrate CPs to form soluble oligosaccharides. Degradation of the oligosaccharides to their constituent sugars or sugar derivatives appears, in many cases, to occur at the cell surface using secreted enzymes with lipoboxes. In those cases where it has been examined, the enzymes with lipoboxes are cell-associated as predicted (e.g., [40]). In this way, most diffusible sugars or sugar derivatives from the CPs would be produced at the cell surface. This argues that the specificity and affinity of the cognate transport systems in the cell membrane would be critical to the ability of this bacterium to utilize these CPs. Little is known of these transport systems.

An example of this strategy is found in the Chi18B chitinase produced by this bacterium. Expression of the source gene is induced by chitin. This dual domain enzyme appears to be secreted and anchored to the outer membrane due to the presence of a lipobox. The catalytic domains are separated from this lipobox by a long polyserine domain (∼110 residues) that would place the enzyme at the outer membrane surface. This placement is augmented by the position of the distinct catalytic domains in which the more distant domain is *endo-*acting forming chitooligosaccharides and the closer domain is *exo-*acting producing chitobiose. Thus the most diffusible products of chitin are formed at the cell surface [47].

Other systems are more complicated as the core polymers are modified. Removal of the modifications (e.g., pectin esterases, acetoxylan esterases, *etc.*) appears to occur at the cell surface in some cases as these enzymes have lipoboxes. This was not anticipated as homologs of many secreted depolymerases only act on the core polymer lacking modifications. This predicts that the depolymerases of this bacterium may lack specificity in their substrate preference (e.g., have the ability to degrade acetylated substrates). Alternatively, the secreted enzymes may be limited in their activity until the surface-associated enzymes act on the material. Thus, close proximity to the adsorbed bacterium would be necessary for these secreted enzymes to be active.

This bacterium has the potential to produce a large number of carbohydrases to degrade the CPs found in the marine environment. Most other bacteria produce substantially fewer carbohydrases and tend to degrade less types of CPs [10]. With such a plethora of enzymes, where did these genes come from? Most likely, these genes were acquired by horizontal gene transfer as their codon usage and the third position nucleotide in codons differ from that used by the core housekeeping genes [10]. The mechanism of acquisition, however, is unclear. Originally, the genes associated with each system were largely thought to be dispersed in the genome [10], and thus, acquired independently. As shown in the Tables, however, genes of several systems are often clustered to a degree. For example, genes encoding components of the agarase system are clustered around genes 1175 and 2650. Similarly, some of the alginolytic system clusters near genes 1270 and 3274. The amylase system is concentrated near gene 556 and the pectinolytic system has a focus near gene 937. These areas of concentration may reflect the evolution of the genes as blocks of genes could have been acquired through horizontal gene transfer. For example, the agarolytic systems genes between 2649 and 2657 show transcriptional and translational similarity to their counterparts in *Pseudoalteromonas atlantica*, and thus, are a candidate to have been acquired as a genetic unit. Alternatively, more classic bacterial evolution could have occurred in which acquired genes are duplicated at nearby locations [59]. On the other hand, it may be that gene fragments representing a domain are acquired or duplicated and then recombined with other fragments to form novel enzymes. This could explain some of the unusual apparent enzyme structures of this bacterium and the mixed phylogenies of domains in enzymes like Chi18C and Chi18B [60].

In conclusion, the carbohydrase systems of this bacterium are providing new insights into the degradation of CPs. In some cases, the enzymes of a system seem to be similar to those of other bacteria. In other cases, new activities are identified to explain oddities in the system. This bacterium, thus, can serve as a paradigm for processing of complex polysaccharides in the marine environment and offers the opportunity for comparative studies with terrestrial systems.

## Figures and Tables

**Table 1.** The agarolytic system of *S. degradans*.

**Enzyme**	**Source gene ^1^**	**Secretion ^2^**	**Catalytic domain ^3^**	**CBM ^3^**	**Size (kDa)**	**Annotated activity ^1^**	**Confirmed activity**
Aga16B	1175	+	GH16	6 (×2) ^4^	64.5	endoagarase	+ ^5^
Aga50D	2644	+	GH50		88.6	exoagarase	+ ^6^
Aga86E	2655	+	GH86	6 (×3)	146.2	exoagarase	+ ^5^
Aga50A	1176	Lipobox	GH50		87.4	likely exoagarase	+ ^7^
Aga86C	2650	Lipobox	GH86		86.2	endoagarase	+ ^5^
AgaT	2649	Inner membrane				putative transporter	
Aga117F	2657	-	GH117		41.6	neoagarobiose hydrolase	+ ^8^

1 As described in [10];

2 As indicated by the presence of a Type II secretion signal or a Lipobox as detected by LipoP 1.0 server;

3 as determined by CAZY [11]; GH, Glycoside hydrolase; CBM, carbohydrate binding domain;

4 As described by [18];

5 from [15];

6 from [16];

7 from [19];

8 as described in [17].

**Table 2.** The predicted alginolytic system of *S. degradans*.

**Enzyme**	**Source gene ^1^**	**Secretion ^2^**	**Catalytic domain ^3^**	**CBM ^3^**	**Size (kDa)**	**Annotated activity ^1^**
Alg2A	3278	+	PL2	16, FA58C	58.5	alginate lyase
Alg6B	3285	+	PL6		83.2	same
Alg17C	3284	+	PL7		81.6	same
Alg7D	2547	+	PL7	32, FA58C	65.4	same
Alg6F	2873	+	PL6		163.1	same
Alg7G	1507	+	PL7	32, FA58C	94.3	same
Alg18J	3272	+	PL18	16, 32	58.5	same
Alg14M	3918	+	PL14		44.2	same
Alg7A	3286	Lipobox	PL7		37.5	same
Alg7E	2478	Lipobox	PL7	32, FA58C	56.3	same
Alg6H	3275	Lipobox	PL6		93.6	same
Alg6I	3274	Lipobox	PL6		57.4	same
Alg7K	2839	Lipobox	PL7		50.5	same
DctM	1268	Inner membrane				transporter
DctQ	1267	Inner membrane				transporter
DctP	1266	Periplasm				transporter
HxuI	950	-				4-deoxy-l-*threo*-5-hexosulose uronate isomerase
HxuK	1280	-				same
	1269	-				2-dehydro-3-deoxygluconate kinase
HxuA	1382	-				2-dehydro-3-deoxyphosphogluconate aldolase
AlgR	1270	-				GntR family regulator

1 As described in [10] or here;

2 As indicated by the presence of a Type II secretion signal or a Lipobox;

3 As determined by CAZY [11]; PL, polysaccharide lyase domain.

**Table 3.** The predicted α-glucan degradation system of *S. degradans*.

**Enzyme**	**Source gene ^1^**	**Secretion ^2^**	**Catalytic domain ^3^**	**CBM ^3^**	**Size (kDa)**	**Annotated activity ^1^**
Amy13A	556	+	GH13		70.1	α-amylase
Amy13B	563	+	GH13		81.6	α-amylase
Amy13C	573	+	GH13		64.6	α-amylase
Gly97A	590	+	GH97		76.6	α-glucosidase
Gly97D	2360	+	GH97		76.4	α-glucosidase
Gly97B	2499	+	GH97		77.0	α-glucosidase
Cbm20A	314	+		20	71.0	Starch binding
PulA	560	Lipobox	pul		151.3	pullananase
PulB	589	Lipobox			77.1	pullananase helper
Amy13D	2938	Lipobox	GH13	20	37.5	α-amylase
GlyNAX	600	Lipobox			87.4	glucan 1,4-α-glucosidase
GlgT	1017	Inner membrane				Glc/Gal transporter
GlkA	1018	-				glucose kinase
Gly13E	601	-	GH13		61.3	α-glucosidase
Suc13F	3210	-	GH13		68.3	sucrose phosphorylase

1 As described in [10];

2 As indicated by the presence of a Type II secretion signal or a Lipobox;

3 As determined by CAZY [11].

**Table 4.** The cellulolytic system of *S. degradans*.

**Enzyme**	**Source gene ^1^**	**Secretion ^2^**	**Catalytic domain ^3^**	**CBM ^3^**	**Size (kDa)**	**Annotated activity ^1^**	**Confirmed activity ^4^**
Cel5A	3003	+	GH5 (×2)	6 (×3)	127.2	endoglucanase	+
Cel5D	2636	+	GH5	2a, 10	65.9	endoglucanase	+
Cel5E	2929	+	GH5	6 (×2)	65.4	endoglucanase	+
Cel5F	1572	+	GH5		42.0	endoglucanase	+
Cel5G	3239	+	GH5	6	67.9	endoglucanase	+
Cel5H	3237	+	GH5	6	66.9	endoglucanase	+
Cel5I	3420	+	GH5	2a, 10	77.2	endoglucanase	+
Cel5J	2494	+	GH5	6 (×2)	65.2	endoglucanase	+
Cel6A	2272	+	GH6	2 (×2)	81.2	endoglucanase	+
Cel9A	636	+	GH9		62.7	endoglucanase	+
Cel9B	649	+	GH9	2a, 10	89.5	endoglucanase	+
Cel5B	2490	Lipobox	GH5	6	60.8	endoglucanase	+
Cel5C	0325	Lipobox	GH5		49.1	endoglucanase	+
Gly5L	2996	Lipobox	GH5	6	93.3	endoglucanase	+ ^5^
Gly5M	3023	Lipobox	GH5	6	94.9	endoglucanase	+ ^5^
Ced3A	2497	Lipobox	GH3		116.0	β-glucosidase	+
Ced3B	0245	Lipobox	GH3		92.9	β-glucosidase	+
Bgl3C	2674	Lipobox	GH3		95.4	β-glucosidase	+
Bgl1A	3603	-	GH1		52.8	β-glucosidase	+
Bgl1B	1394	-	GH1		49.8	β-glucosidase	+
Cep94A	1318	-	GH94		91.7	cellobiose phosphorolase	+

1 As described in [10];

2 As indicated by the presence of a Type II secretion signal or a Lipobox;

3 As determined by the CAZY team [11];

4 see [38–40];

5 see [41].

**Table 5.** The chitinolytic system of *S. degradans*.

**Enzyme**	**Source gene ^1^**	**Secretion ^2^**	**Catalytic domain ^3^**	**CBM ^3^**	**Size (kDa)**	**Annotated activity ^1^**	**Confirmed activity**
Chi18A	1704	+	GH18	5	56.3	endochitinase	+ ^4^
Chi18C	3605	+	GH18	5	82.5	endochitinase	+ ^4^
Cdx18A	3902	+	GH18	5	122.1	chitodextrinase	+ ^4^
CbpA	633	+		2, 33	46.1	chitin binding	
Chi18B	3870	Lipobox	GH18 (×2)		135.2	endo/exochitinase	+ ^5^
Hex20A	3037	Lipobox	GH20	FA58C	88.5	*N-*acetylglucosaminidase	+ ^4^
Hex20B	3271	Periplasm	GH20	FA58C	98.4	*N-*acetylglucosaminidase	+ ^4^
NagE	3038	Inner membrane			47.5	NAG transporter	
Hex3C	1790	-	GH3		37.5	*N-*acetylglucosaminidase	+ ^4^
NagF	3036	-			31.8	*N*-acetylglucosamine kinase	
NagC	3047	-			41.4	LacI homolog	
NagB	3041	-			37.0	fructose 6P transaminase	
NagA	3040	-			41.4	*N*-acetylglucosamine deacetylase	

1 As described in [10];

2 As indicated by the presence of a Type II secretion signal or a Lipobox;

3 As determined by CAZY [11];

4 As described in [41];

5 As described in [47].

**Table 6.** The predicted laminarinase system of *S. degradans*.

**Enzyme**	**Source gene ^1^**	**Secretion ^2^**	**Catalytic domain ^3^**	**CBM ^3^**	**Size (kDa)**	**Annotated activity ^1^**
Lam16A	1393	+	GH16	6 (×2), 56	183.2	β-1,3(4)-endoglucanase
Lam16B	2927	+	GH16	6, FA58C (×2)	158.6	β-1,3(4)-endoglucanase
Lam16C	1444	+	GH16	4, 32	129.1	β-1,3(4)-endoglucanase
Lam16D	3021	+		FA58C	77.7	β-1,3(4)-endoglucanase
Lam16E	0652	+	GH16	6 (×2)	61.4	β-1,3(4)-endoglucanase
Gly16H	2878	+	GH16	32, FA58C	107.2	β-1,3(4)-endoglucanase
Lam16F	3141	Lipobox	GH16		80.2	β-1,3(4)-endoglucanase
Lam16G	2832	Lipobox	GH16	6 (×2)	94.2	β-1,3(4)-endoglucanase
Lam81A	2834	Lipobox	GH16	56	133.1	β-1,3-endoglucanase
Gly30A	2992	Lipobox	GH30	6 (×2), 13	107.4	β-1,6-glucosidase
Gly30B	2994	Lipobox	GH30		52.8	β-1,6-glucosidase
Lam55A	54	-	GH55	FA58C	58.5	β-1,3-endo/exoglucanase

1 As described in [10];

2 As indicated by the presence of a Type II secretion signal or a Lipobox;

3 As determined by CAZY [11].

**Table 7.** The predicted mannanase system of *S. degradans*.

**Enzyme**	**Source gene ^1^**	**Secretion ^2^**	**Catalytic domain ^3^**	**CBM ^3^**	**Size (kDa)**	**Annotated activity ^1^**
Man5O	656	+	GH5	10	52.6	mannanase
Man5N	64	+	GH5	2, 10	57.6	mannosidase
Gly5K	2993	+	GH5	6 (×2), 13	94.6	
Man5P	509	Lipobox	GH5		50.9	mannanase
ManR	2285	Lipobox			42.8	mannanase
Man5Q	2541	Lipobox	GH5		92.3	mannanase
Man26A	3691	Lipobox	GH26	10, cbm ^4^	54.9	mannosidase
Man2A	169	-	GH2		91.7	mannosidase
Man2B (Gly5R)	1121	-	GH2		59.2	mannanase

1 As described in [10];

2 As indicated by the presence of a Type II secretion signal or a Lipobox;

3 As determined by CAZY [11];

4 not currently classified CBM.

**Table 8.** The probable pectinase system of *S. degradans*.

**Enzyme**	**Source gene ^1^**	**Secretion ^2^**	**Catalytic domain ^3^**	**CBM ^3^**	**Size (kDa)**	**Annotated activity ^1^**
Pel1B	937	+	PL1		46.1	pectate lyase
Pel1C	942	+	PL1	6, 35	78.9	pectate lyase
Pel1A	943	+	PL1	6, 35	136.4	pectate lyase
Pel1F	2311	+	PL1	2, 6, 35	81.5	pectate lyase
Pel3B	608	+	PL3		46.1	pectate lyase
Pel3D	1703	+	PL3	13	41.6	pectate lyase
Pel3A	2308	+	PL3	2, 6	52.3	pectate lyase
Pel3C	3007	+	PL3		42.9	pectate lyase
Pel10A	1051	+	PL10	2, 6, 35	73.3	pectate lyase
Pel11A	1650	+	PL11	35	97.6	rhamnogalacturon lyase
Rgh105B	2808	+	GH105		45.5	rhamogalacturon hydrolase
Rgh105C	3946	+	GH105		44.3	rhamnogalacturon hydrolase
Rgh105A	951	Lipobox	GH105		92.4	rhamnogalacturon hydrolase
PelG	3881	Lipobox	Pel	6	78.2	pectate lyase
PmeA	890	Lipobox			75.9	pectinesterase
Pme12A	3094	Lipobox	CE12		30.2	pectinesterase
Pel43X	944	Lipobox	GH43		42.4	pectinesterase
PmeB	3447	Lipobox			115.1	pectinesterase
Pel1D	3448	Lipobox	PL1		63.3	pectinesterase
Pel1E	2307	Lipobox	PL1		45.4	pectate lyase
Pel9A	2946	Lipobox	PL9		75.6	exopectate lyase
Pel10B	2947	Lipobox	PL10	6, 35	60.8	pectate lyase

1 As described in [10];

2 As indicated by the presence of a Type II secretion signal or a Lipobox;

3 As determined by CAZY [11].

**Table 9.** The xylanolytic system of *S. degradans*.

**Enzyme**	**Source gene ^1^**	**Secretion ^2^**	**Catalytic domain ^3^**	**CBM ^3^**	**Size (kDa)**	**Annotated activity ^1^**	**Confirmed activity**
Xyn10A	181	+	GH10	5, 7	61.7	endoxylanase	+
Xyn10B	2934	+	GH10	2,10	65.0	endoxylanase	+
Xyn10C	2633	+	GH10		92.3	endoxylanase	+
Xyn10D	3612	+	GH10, GH43	2, 6, 22	129.6	endoxylanase	+ ^4^
Xyn11A	701	+	GH11			endoxylanase	+
Xyn11B	3061	+	GH11, CE11	2, 10, 60	80.8	endoxylanase	+
Xyl43J (Arg43J)	789	+	GH43		35.1	xylosidase	
Xyl43L	946	+	GH43		36.1	xylosidase	
Gal31A	1593	+	GH31		46.5	α-galactosidase	
Glu115A	1755	+	GH115		110.0	α-glucuronidase	
Glu2G	2632	+	GH2		68.8	β-glucuronidase	
Arb51A	1767	+	GH51		59.3	arabinofuranosidase	
Arb43A	2809	+	GH43	6, 13, 35	85.0	arabinofuranosidase	
Arb43D	1014	+	GH43	13	92.4	arabitan *endo* 1,5-α-arabinosidase	
Arg53B	3710	+	GH53	13	52.6	arabinogalactan *endo* 1,4-β-galactosidase	
Axe2A	2370	+	CE2		40.9	acetoxylan esterase	
Gal2B	1177	+	GH2		97.1	β-galactosidase	
Gal2F	3882	+	GH2	35	107.1	β-galactosidase	
Xyn10E	323	Lipobox	GH10		75.2	endoxylanase	+
Arb43H	598	Lipobox	GH43		63.8	arabinofuranosidase	
Arb43B	787	Lipobox	GH43		40.7	arabinofuranosidase	
Gly97C	790	Lipobox	GH97		73.3	α-glucosidase	
Arb43I	1655	Lipobox	GH43		62.6	arabinofuranosidase	
Arb43J	791	Lipobox	GH43	13	67.2	arabinofuranosidase	
Arb43K	822	Lipobox	GH43		42.6	xylosidase	
Xyl31A	2500	Lipobox	GH31		110.2	xylosidase	
Arg53A	683	Lipobox	GH53			arabinogalactan *endo* 1,4-β-galactosidase	
Arg53C	2827	Lipobox	GH53	Ricin	71.1	arabinogalactan *endo* 1,4-β-galactosidase	
Gly43M	3317	Lipobox	GH43		127.2	arabinofuranosidase	
Arb43E	786	Lipobox	GH43			arabitan *endo* 1,5-α-arabinosidase	
Axe1C	3746	Lipobox	CE1		37.0	acetoxylan esterase	
Axe2C	3143	Lipobox	CE2		40.4	acetoxylan esterase	
Axe3A	3994	Lipobox	CE3		27.8	acetoxylan esterase	
Gal2A	684	Lipobox	GH2		88.1	β-galactosidase	
Gal2C	1285	Lipobox	GH2		91.8	β-galactosidase	
Gal2E	2936	Lipobox	GH2		34.0	β-galactosidase	
Gal2D	2935	-	GH2		65.6	β-galactosidase	
Arb43C	777	-	GH43		35.9	arabinofuranosidase	
Agu67A	1025	-	GH67		89.1	α-glucuronidase	
	51	-	CE1		30.5	carboxyl esterase	
	2890	-	CE1		30.6	carboxyl esterase	
	139	-	CE4		34.3	carboxyl esterase	
	653	-	CE4		41.2	carboxyl esterase	

1 As described in [10];

2 As indicated by the presence of a Type II secretion signal or a Lipobox;

3 As determined by CAZY [11];

4 As shown by Ko *et al.* [56].
